# Pushing the Limits: Chronotype and Time of Day Modulate Working Memory-Dependent Cerebral Activity

**DOI:** 10.3389/fneur.2015.00199

**Published:** 2015-09-25

**Authors:** Christina Schmidt, Fabienne Collette, Carolin F. Reichert, Micheline Maire, Gilles Vandewalle, Philippe Peigneux, Christian Cajochen

**Affiliations:** ^1^Cyclotron Research Centre, University of Liège, Liège, Belgium; ^2^Neuropsychology Unit, University of Liège, Liège, Belgium; ^3^Centre for Chronobiology, Psychiatric Hospital of the University of Basel, Basel, Switzerland; ^4^UR2NF – Neuropsychology and Functional Neuroimaging Research Unit affiliated at CRCN – Center for Research in Cognition and Neurosciences, Neurosciences Institute, Université Libre de Bruxelles, Brussels, Belgium

**Keywords:** chronotype, working memory, time of day, BOLD activity, task complexity

## Abstract

Morning-type individuals experience more difficulties to maintain optimal attentional performance throughout a normal waking day than evening types. However, time-of-day modulations may differ across cognitive domains. Using functional magnetic resonance imaging (fMRI), we investigated how chronotype and time of day interact with working memory at different levels of cognitive load/complexity in a N-back paradigm (N0-, N2-, and N3-back levels). Extreme morning- and evening-type individuals underwent two fMRI sessions during N-back performance, one 1.5 h (morning) and one 10.5 h (evening) after wake-up time scheduled according to their habitual sleep–wake preference. At the behavioral level, increasing working memory load resulted in lower accuracy while chronotype and time of day only exerted a marginal impact on performance. Analyses of neuroimaging data disclosed an interaction between chronotype, time of day, and the modulation of cerebral activity by working memory load in the thalamus and in the middle frontal cortex. In the subjective evening hours, evening types exhibited higher thalamic activity than morning types at the highest working memory load condition only (N3-back). Conversely, morning-type individuals exhibited higher activity than evening-type participants in the middle frontal gyrus during the morning session in the N3-back condition. Our data emphasize interindividual differences in time-of-day preferences and underlying cerebral activity, which should be taken into account when investigating vigilance state effects in task-related brain activity. These results support the hypothesis that higher task complexity leads to a chronotype-dependent increase in thalamic and frontal brain activity, permitting stabilization of working memory performance across the day.

## Introduction

Many factors contribute to daily decisions about when to go to bed and when to get up. Beside social and socioprofessional timing constraints, interindividual differences in the regulation of sleep and wakefulness states exert a noticeable influence on these decisions as well as on the optimal timing to perform cognitively demanding tasks. The propensity to be asleep and to be awake at specific time windows over the 24 h light–dark cycle defines the particular chronotype of an individual (1). Extreme morning chronotypes are at one end of the continuum. They exhibit preference for waking up very early in the morning and find it difficult to remain awake beyond their usual bedtime (1). At the opposite end of the continuum, extreme evening types strongly prefer to go to bed at late hours of the night and find it difficult to get up early in the morning. Extreme chronotypes exhibit a phase-shifted circadian rhythmicity in such a way that maximal and minimal values of physiological circadian markers (e.g., core body temperature and melatonin) occur earlier or later (2–7). Accumulating evidence also suggests that homeostatic sleep regulation differs between chronotypes. As quantified by electroencephalographic theta activity during wakefulness (6.25–9 Hz) and slow wave activity (SWA) during NREM sleep (1–5 Hz), homeostatic sleep pressure builds up (8) and dissipates (9–11) faster in morning than evening chronotypes.

Interactions between circadian and homeostatic factors result in time-of-day modulations of behavioral performance as well as of spontaneous and evoked brain activity patterns. That time of day influences regional brain activity was already highlighted in a positron emission tomography (PET) study (12). Glucose metabolism increased in evening when compared with morning wakefulness sessions in hypothalamic and brainstem areas putatively implicated in arousal promotion. Conversely, temporal and occipital cortices exhibited decreased metabolism in the evening. More recent functional magnetic resonance imaging (fMRI) studies have explored time-of-day modulations of brain activity underlying cognitive performance [see Ref. (13) for a review]. Time-of-day modulation of blood oxygen level-dependent (BOLD) responses on interfering items of a Stroop-like Color-Word task was found in brain regions associated with attentional orienting and executive control systems such as the parietal lobe and the frontal eye field (14). In most brain areas, stimulus-related BOLD responses decreased from morning (6:00 a.m.) to evening (6:00 p.m.) hours, except at the latest time point (around 10:00 p.m.) during which activity increased again (14). Additionally, individual differences in chronotype markedly influence time-of-day modulation on cerebral activity patterns supporting cognitive performance. For instance, late chronotypes also reach maximal finger tapping-related neural activity in the supplementary motor area, parietal cortex, and rolandic operculum latest in the day (15). These results indicate that morningness–eveningness traits interact with time of day to modulate regional cerebral activity supporting motor skills. In the attentional domain, we reported higher BOLD responses in the evening hours in extreme evening, compared to morning chronotypes in brain sites compatible with key arousal modulation structures [locus coeruleus (LC) and suprachiasmatic area (SCA) (11)] during a psychomotor vigilance task (PVT) (16). Finally, in a Stroop task, morning types exhibited decreased task-related BOLD responses from morning to evening hours in brain areas supporting cognitive interference (e.g., insula and cingulate cortex), whereas evening chronotypes exhibited the reversed pattern (17).

Cognitive load and task complexity are interacting with lack of sleep to modulate brain activity patterns supporting cognitive performance (18). It was proposed that sleep loss-induced deteriorations of performance at greatest task complexity can be minimized/compensated by temporarily increasing prefrontal and thalamic activation (19). Cognitive tasks featuring parametric variations of working memory load are well adapted to test this hypothesis. The N-back task (20) is paradigmatic in this context because task complexity can be modulated by simply changing the number of elements (i.e., the size of *N*) that must be kept in memory to detect identical items within a continuous series. Increasing working memory load in the N-back is classically associated with increased prefrontal activation (21). Notwithstanding, prefrontal activation may saturate or even decline when reaching the highest load levels (22), a result interpreted as supporting evidence for a capacity-constrained working memory system. Finally, Choo et al. (23) disclosed interactions between vigilance states (i.e., normally rested vs. sleep deprived) and the working memory load-dependent modulation of neural activity in the left prefrontal cortex and thalamus during performance on an N-back task.

Given the combined impact of vigilance states and task complexity on performance and underlying brain responses, we posited in the present study that morningness–eveningness should interact with modulation of brain activity by time of day and task complexity as manipulated by working memory load. To test this hypothesis, extreme morning and evening chronotypes were administered a three-level N-back task (N0-back, N2-back, and N3-back) during two fMRI sessions scheduled in the participants’ subjective morning and evening hours. Morning and evening hours were adapted to the habitual sleep–wake schedule preferences of each individual (1.5 and 10.5 h after preferred wake-up time). We predicted that activity in working memory load-sensitive brain areas (20, 21, 23) will be modulated by both chronotype and time of day. We expected more distinctive effects of time of day and chronotype on cerebral activity at high working memory load.

## Materials and Methods

### Participants

Thirty-two young healthy volunteers [16 extreme morning (MT), 16 extreme evening (ET) types] gave written informed consent to participate in this study approved by the institutional Ethics Committee. Individuals reporting medical, psychiatric, or sleep disorders were excluded. Further exclusion criteria comprised medication or drug consumption, alcohol abuse, excessive caffeine consumption or physical activity, shift work, or flights passing more than two time zones within the past 3 months. Subjects were screened for their sleep–wake timing preferences using the morningness–eveningness questionnaire (MEQ) (1). Scores >70 index extreme morning types and scores <30 index extreme evening types. Two morning- and three evening-type volunteers performed at chance levels during the N3-back condition (<57% of correct responses, >2 SD of overall mean); they were excluded for the analysis because adequate task engagement could not be guaranteed. The two groups matched according to age (MT 24.4 ± 2.3 vs. ET 24.8 ± 4.9 years) and educational level and did not differ in anxiety and depression levels (Beck Depression and Anxiety Inventory) (24, 25) as well as sleep quality (Pittsburgh Sleep Quality Index) (26) and daytime sleepiness (Epworth sleepiness scale) (27) scores (all *p*s > 0.12).

### Procedures

Individual sleep schedules were determined according to the volunteer’s preferred sleep and wake timing, with the constraint that they were required to sleep 8 h. Screened subjects entered the sleep facility for a habituation night. After this night, they were asked to follow the sleep schedule (±30 min) they would spontaneously adopt while free from any social constraints but to keep their bedtime duration at 8 (±1) h for the week preceding the laboratory part of the study. Compliance to the selected rest-activity patterns was assessed using sleep logs and continuous actimetric recordings of motor activity of the non-dominant arm (Cambridge Neurotechnologies, UK) the week prior the experimental session. Subjects then entered the sleep laboratory for two nights. The precise schedule of each session was individually adapted to the subject’s habitual bedtime. They came to the laboratory 7 h before habitual lights off on day 1. After the hook-up of the electrodes, subjects continuously stayed under controlled semi-recumbent posture and food intake conditions in dim light (<10 lux), except for the sleep episode where they were lying in horizontal position in bed in complete darkness (0 lux). These conditions aimed at controlling for modulatory effects of external alerting cues (i.e., light history, physical activity, and consumption of stimulants) on chronotype-dependent time-of-day effects on salivary melatonin, cognitive performance, and subjective sleepiness measures. Subjective sleepiness [visual analogue scale (VAS) and Karolinska Sleepiness scale (KSS)] (27) and objective vigilance (the PVT) (16) were assessed at hourly intervals while awake and saliva samples were hourly collected to assay melatonin. Polysomnographic data (Fz, Cz, Pz, Oz, EOG, EMG) recorded during the night were reported elsewhere (11). After lights off, subjects were allowed to sleep for 8 h. One and a half [morning session (MS)] and 10.5 h [evening session (ES)] after wake up at the scheduled timing, subjects underwent a fMRI session during which they were administered three cognitive tasks in counterbalanced order across subjects and sessions. Half of ET and MT participants had their morning fMRI session after the first experimental night and the fMRI ES AFTER the second one. For the other half, the evening fMRI session followed the first experimental night and the fMRI MS followed the second one.

Here, we report results related to the N-back task. Results related to the PVT and the Stroop tests in the same study protocol are reported elsewhere (11, 17).

### N-back paradigm

Experimental stimuli consisted of pseudorandomized sequences of phonologically dissimilar consonants printed in gray color on a black screen. In the N-back task, subjects have to indicate using a handheld response box whether the displayed letter matches the stimulus presented *n* trials ago (*n*-back level 2 or 3). In the 0-back condition, they had to indicate whether the current stimulus matched the predetermined letter “K.” In all three conditions [0-back (N0), 2-back (N2), and 3-back (N3)], targets were presented in 33% of the trials. Each session consisted of five blocks for each condition (15 blocks in total) presented in pseudorandomized order (maximum two blocks of the same condition successively). Each block consisted of 33 trials (interstimulus interval, 2,000 ms). Prior to each block, a cue indicating the condition to be performed appeared for 5,000 ms. Before the first scanning session, subjects were familiarized with the task. Time of day and chronotype modulations in N-back performance were assessed using repeated-measures ANOVA on accuracy measures (hit targets, i.e., correct “yes” answers, corrected for false alarms, i.e., wrong “yes” answers) including the within factors “working memory load” (0-, 2-, 3-back) and “testing time” (morning, evening) and the group factor “chronotype” (morning types, evening types).

### fMRI data acquisition

Functional magnetic resonance imaging series were acquired using a head-only 3T scanner (Siemens, Allegra, Erlangen, Germany). Multislice T2*-weighted fMRI images were obtained with a gradient echo-planar sequence using axial slice orientation (TR = 2,130 ms, TE = 40 ms, FA = 90°, 32 transverse slices, 3 mm slice thickness, 30% interslice gap, FoV = 220 mm × 220 mm, matrix size = 64 × 64 × 32, voxel size = 3.4 mm × 3.4 mm × 3.0 mm). The three initial scans were discarded to avoid T1 saturation effects. For anatomical reference, a high-resolution T1-weighted image was acquired [3D MDEFT (28); repetition time = 7.92 ms, echo time = 2.4 ms, inversion time = 910 ms, flip angle = 15°, field of view = 256 mm × 224 mm, matrix size = 256 × 224 × 176, voxel size = 1 mm × 1 mm × 1 mm].

### fMRI data analysis

Functional magnetic resonance imaging data from MSs and ESs were analyzed using Statistical Parametric Mapping 5 (SPM5; http://www.fil.ion.ucl.ac.uk) implemented in MATLAB 7 (Mathworks, Sherborn, MA, USA). Functional scans were realigned using iterative rigid body transformations that minimize the residual sum of square between the first and subsequent images. They were then normalized to the Montreal Neurological Institute (MNI) EPI template (two-dimensional spline; voxel size, 2 × 2 × 2) and spatially smoothed with a Gaussian kernel with full width at half-maximum (FWHM) of 8 mm. Data were processed using two-step analyses taking into account intraindividual variance than interindividual variance. For each subject, changes in regional brain responses were estimated using a general linear model, in which the blocks in N0-, N2-, and N3-back conditions were modeled using boxcar functions and convolved with a canonical hemodynamic response function. Motion regressors derived from realignment of the functional volumes (three translations and three rotations) were considered as covariates of no interest. High-pass filtering was implemented in the matrix design using a cutoff period of 256 s to remove low-frequency drifts from the time series. Effects of interest were tested by linear contrasts at the individual level, generating statistical parametric maps. Contrasts of interest included the main effects of working memory load (N3 vs. N0, N2 vs. N0, and N3 vs. N2) and their interaction with time of day (MS vs. ES). The resulting summary statistic images (one per contrast per subject) were then entered in a second-level analysis accounting for between-subjects variance in the effects of interest (random effects model). Two-sample *t* tests (MT vs. ET) were computed for each contrast of interest to assess the hypothesis that chronotype and working memory load affects time-of-day-dependent BOLD activity in task-related regions. Hence, we computed an interaction between chronotype, time of day, and working memory load [e.g., (N3 vs. N0) × (MS vs. ES) × (MT vs. ET)]. The same procedure was applied for N2 vs. N0 and N3 vs. N2 contrasts. Statistical inferences were performed after family-wise error (FWE) correction for multiple comparisons at a threshold of *p* = 0.05 based on the Gaussian random field theory and computed on the entire brain volume (main effects of task condition). For interaction effects, statistical inferences were performed after correction within small spherical volumes (10 mm radius) around *a priori* locations of activation in working memory load-sensitive brain areas derived from the literature [i.e., thalamus (8 14 4) and middle frontal gyrus (32 42 10)] (20).

## Results

### N-back performance

#### Accuracy

**Figure 1:**
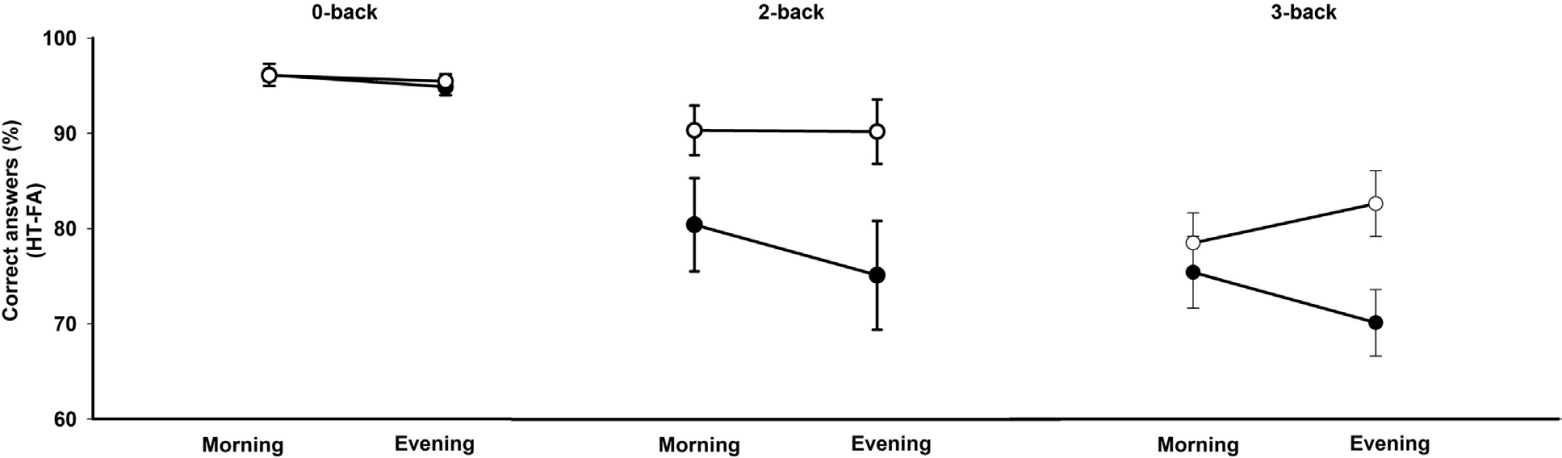
**Accuracy scores (percentage of correct responses minus false alarms) in the N-back task according to the working memory load condition (0-, 2-, 3-back), the time of day (morning, evening), and the chronotype (morning type, evening type)**. Filled circles: morning types; open circles: evening types.

A repeated-measures ANOVA was conducted on accuracy scores (i.e., hits minus false alarms) with within-subject factors working memory condition and time of day and between-subjects factor chronotype (Figure 1). This analysis revealed a main effect of the working memory condition [*F*(2,52) = 83.48, *p* < 0.00001]. Participants performed better on the 0-back than on the 2-and 3-back conditions and better on the 2-back than on the 3-back condition (all *p*s < 0.001). There was also a main effect of chronotype [*F*(1,26) = 4.54, *p* < 0.05] with evening types performing better than morning types irrespective of the condition. The main effect of time of day was not significant [*F*(1,26) = 0.51, *p* = 0.48]. The interaction effects between chronotype and time of day [*F*(1,26) = 2.72, *p* = 0.11] and between chronotype, time of day, and task condition [*F*(2,52) = 2.34, *p* = 0.10] did not reach significance. If considering performance on the N3-back condition (vs. 0-back) separately, there was a significant interaction between chronotype and time of day [*F*(1,26) = 4.22, *p* = 0.05]. Evening types performed better than the morning types in the evening in the 3-back condition (*p* < 0.05). Interaction effects between chronotype and time of day failed to reach significance either considering 2-back vs. 0-back or 3-back vs. 0-back (all *p*s < 0.1).

#### Reaction Times

A repeated-measures ANOVA was computed on reaction times for correct responses with within-subject factors working memory condition and time of day, and between-subjects factor chronotype. This analysis revealed a main effect of the working memory condition [*F*(2,52) = 108.6, *p* < 0.0001], with participants performing faster on the 0-back than on the 3-and 2-back as well as faster on the 2-back compared with the 3-back. There was also a trend for a main effect of chronotype [*F*(1,26) = 3.7, *p* = 0.06], with morning types exhibiting slower reaction times than evening types, independent of time of day and working memory load condition. The main effect of time of day was not significant [*F*(1,26) = 1.9, *p* = 0.18]. The interaction effects between chronotype and time of day [*F*(1,26) = 1.7, *p* = 0.21] and between chronotype, time of day, and task condition [*F*(2,52) = 1.4, *p* = 0.25] did not reach significance. Separate analyses of variance comparing 3- vs. 0-back and 2- vs. 0-back separately revealed, besides a main effect of task complexity, a main effect of chronotype [*F*(1,26) = 4.79, *p* < 0.05], with morning types exhibiting slower reaction times than evening types. A similar pattern was observed when comparing 0-back with the 2-back condition (main effect of task condition and main effect of chronotype: all *p*s < 0.05) and the 2-back with the 3-back condition (main effect of chronotype and main effect of time of day: all *p*s < 0.05).

### fMRI analyses

#### Main Effect of Cognitive Load

When compared with the N0-back condition, BOLD responses were higher in the N2-and N3-back conditions in a distributed network encompassing the inferior frontal gyrus extending into the middle frontal gyrus, the anterior cingulate and the inferior parietal cortex, the insula, parts of the cerebellum, and the thalamus (Table 1). Activity in the insula was higher in the N3-back than in the N2-back condition. In all other areas, activity was not increased in N3-back compared with N2-back condition, indicating a capacity-constrained pattern of activation (FWE corrected over the entire brain volume).

**Table 1:** **Regions where activity was significantly modulated by the task condition**.

Brain area	Side	*Z*-score	*P*_FWE_	*x*	*y*	*z*
**Areas with greater activity during 3-back blocks compared with 0-back blocks (N3>N0)**						
Inferior frontal	L	6.96	<0.0001	−48	10	32
Middle frontal	R	7.02	<0.0001	44	35	24
	R	5.58	<0.0001	32	5	52
	L	5.39	<0.005	−36	52	8
		4.62	<0.05	−24	46	6
Anterior cingulate	L	7.11	<0.0001	−5	24	46
Inferior parietal	R	7.14	<0.0001	52	−48	50
	L	7.04	<0.0001	−30	−58	50
	R	6.96	<0.0001	34	−58	48
Precuneus	R	5.80	<0.0001	10	−72	54
Insula	R	7.59	<0.0001	32	24	−4
	L	6.69	<0.0001	−28	22	2
Cerebellum	R	5.40	<0.005	34	−62	−36
	L	5.32	<0.005	−28	−64	−36
Thalamus	R	4.94	<0.05	10	−18	10
	R	4.85	<0.05	16	0	12
	L	4.78	<0.05	−10	−16	10
**Areas with greater activity during 2-back blocks compared with 0-back blocks (N2>N0)**						
Inferior frontal	L	7.24	<0.0001	−44	6	32
Middle frontal	R	7.42	<0.0001	30	8	54
	L	5.5	<0.005	−34	54	22
Anterior cingulate	L	11.07	<0.0001	−8	20	50
Inferior parietal	L	8.14	<0.0001	−30	−58	50
Insula	R	7.82	<0.0001	32	24	−4
	L	7.35	<0.0001	−30	22	4
Inferior temporal	R	5.05	<0.05	58	−50	−14
Cerebellum	R	5.40	<0.005	44	−66	−32
	L	5.32	<0.005	−28	−64	−36
Thalamus	L	5.59	<0.005	−10	−16	12
	R	5.59	<0.005	10	−16	10
**Areas with greater activity during 3-back blocks compared with 2-back blocks (N3>N2)**						
Insula	R	3.21	<0.05	42	32	38

*Coordinates (x, y, z) are expressed in millimeters in the Montreal Neurological Institute (MNI) space. P_FWE_: correction for multiple comparisons at a threshold of p = 0.05 based on the Gaussian random field theory and computed on the entire brain volume. R = right, L = left*.

### Main effect of time of day and chronotype

When comparing 2-back with the 0-back condition, a main effect of time of day was observed in the ventrolateral prefrontal cortex [−40 24 28] [*Z*-score = 3.48, *p*_svc_ = 0.011, small volume correction according to coordinates taken from Ref. (20)] such that, independently of chronotype, BOLD activity decreased from morning to evening hours in the 2-back condition only. For the comparison between 3- and 0-back conditions, a main effect of time of day was detected in the lateral premotor area [26 2 58] [*Z*-score = 3.42, *p*_svc_ = 0.015, small volume correction according to coordinates taken from Ref. (20)]. Activity in this region increased from the morning to the evening hours in the 0-back condition, whereas it decreased throughout the day during performance in the 3-back condition.

No significant main effect of chronotype on BOLD activity was evidenced when considering correction for multiple comparisons including ROIs within the main effect of task load (N3>N0), (N2>N0), or (N3>N2), respectively, or when using small volumes of interest according to Ref. (20).

### Modulation of task load-related activation by time of day and chronotype

**Figure 2:**
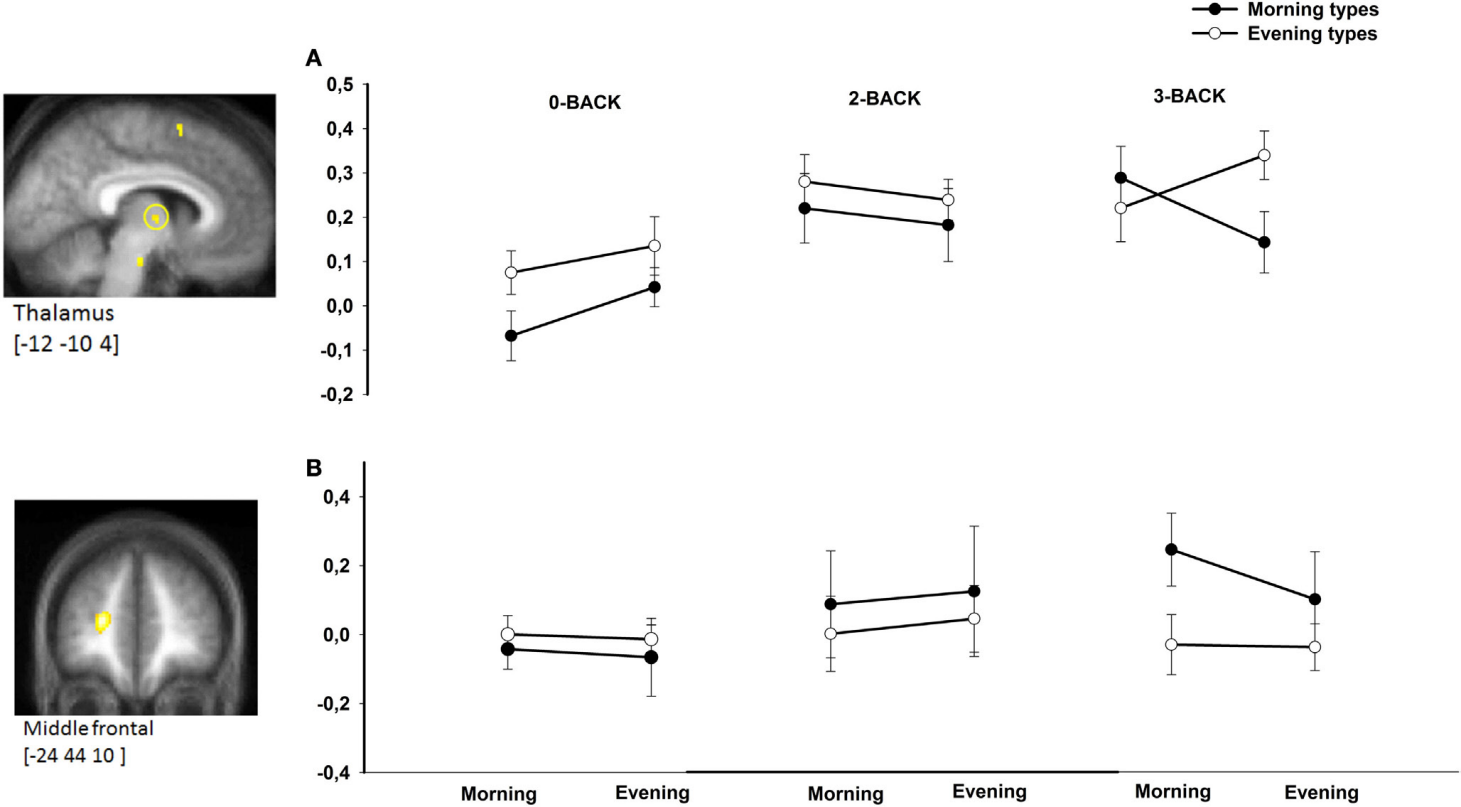
**BOLD responses in the thalamus (A) and middle frontal gyrus (B) in morning compared with evening types during subjective morning and evening sessions according to working memory load (N0, N2, N3)**. Contrasts are displayed at *p* < 0.001, uncorrected threshold, overlaid on the mean normalized structural MR image of the population (*n* = 28). Corresponding parameter estimates are plotted in the right (arbitrary units).

There was no triple interaction effect (chronotype × time of day × N-back level) on BOLD responses in *a priori* defined areas using the N2>N0 comparison. However, task-related BOLD activity was significantly modulated by chronotype and time of day in the left middle frontal gyrus ([−24 48 10], *Z*-score = 4.71, *p*_svc_ = 0.042) and in the thalamus ([−12 −10 4], *Z*-score = 3.21, *p*_svc_ = 0.049) in the N3>N0 condition comparison as well in the N3>N2 condition comparison (Figure 2). Note that both regions were also more activated in the 3-back compared with the 0-back condition (*p* < 0.05, FWE corrected over the entire brain volume, included within the cluster of middle frontal gyrus and thalamus described in Table 1). Furthermore, both regions were previously reported as task load-sensitive brain areas in the literature (20). Overall, *post hoc* analyses disclosed significant differences between chronotypes during the subjective morning or the subjective evening at the highest working memory load condition (3-back) only. When compared with morning types, evening types exhibited higher thalamic BOLD responses in the N3 (vs. N2 or N0) condition in the evening hours, whereas morning types had higher BOLD responses than evening types in the morning hours in the middle frontal gyrus.

## Discussion

We administered a working memory N-back task including three levels of complexity/cognitive load after 1.5 and 10.5 h of wakefulness to extreme morning and evening chronotype participants who lived according to their preferred sleep–wake schedule for the duration of 1 week. In line with our predictions, the fMRI data analysis showed that chronotype and time of day interacted with the working memory load-related modulation of BOLD activity in cortical and thalamic areas throughout a normal waking day, particularly at highest cognitive load levels.

Several studies reported highest performance in executive control tasks in the morning for morning types and in the evening for evening types. These synchrony effects (29) are mainly observed on difficult task conditions requiring controlled processing (30–32). Here, we assessed working memory and its underlying cerebral correlates using a visual verbal N-back task. Successful manipulation of information in short-term storage is the main process characterizing working memory performance (33). Only when considering the highest cognitive load (i.e., N3-back condition), performance in evening types improved from morning to evening hours and was significantly higher than in morning types during evening hours. Hence, behavioral trends observed at highest levels of complexity are comparable with synchrony effects reported in previous studies (30).

The neural correlates of working memory processes have been frequently investigated in neuroimaging studies using the visual verbal N-back task (20, 34). Performance in this task is underpinned by activity in a wide set of brain regions encompassing prefrontal and parietal regions as well as the occipital lobe, the thalamus, and the cerebellum. Each of these structures was linked to several functional aspects of working memory (35). Among those, prefrontal activity has been associated with the limited capacity to handle information (22), a fundamental aspect of working memory. Prefrontal activation increases monotonically with task load (21) but can also peak and then decline following an inverted U-shape (22), suggesting that the working memory system has a constrained capacity. In the study by Callicott et al. (22), activity in dorsolateral prefrontal cortex areas followed this inverted U-shaped pattern from lowest to highest working memory load, whereas activity in other brain regions often reached an earlier plateau or exhibited continuously increasing BOLD activity. Likewise, Choo et al. (23) observed increasing left prefrontal activation from the N1-back to the N2-back condition, which did not further increase or even exhibited a trend toward a decrease at the N3-back level. Similarly, we found significantly increased activity from the N0- to the N2-back condition in task load-sensitive brain regions, but no further increase from the N2- to the N3-back condition, except in the insula. Thus, our findings are in accordance with the suggestion that working memory has limited capacity, which is mirrored at the cerebral level.

Extreme morning and evening chronotypes differ in homeostatic sleep–wake regulation (9–11, 36). In a previous study (11), we found increased levels of majorly homeostatically regulated sleep SWA at the beginning of the night in morning-type individuals compared with evening-type individuals. In parallel, at the behavioral level, morning types exhibited higher subjective sleepiness and lower objective vigilance in the evening hours compared with the evening types. Furthermore, during a vigilance task probing a fundamental form of attention on which many other cognitive processes build on (37), optimal performance maintenance in the subjective evening hours was associated with higher activity in evening than morning chronotypes in a region comprising the LC and in an anterior hypothalamic region putatively encompassing the SCA (11). Both LC and SCA are involved in the generation of the circadian arousal signal (38). These results supported the assumption that the evening circadian alerting signal is acting less powerfully in morning than in evening types, either due to or leading to disproportionally increased homeostatic sleep pressure in morning types. How this hypothesis can be translated into the context of a working memory paradigm has not been explored to date. Notwithstanding, many neuroimaging studies make use of total sleep deprivation protocols to investigate the impact of increased homeostatic sleep pressure on working memory-related brain activity. Sleep loss-related decreased activity was mainly observed in the fronto–parieto–occipital network in association with decreasing working memory performance (19, 23, 39–43). Alongside, successful maintenance of stable working memory performance in a sleep-deprived state was related to increased compensatory activity in frontal, anterior cingulate, and thalamic areas (19, 23, 44). Task complexity may additionally modulate compensatory increases in brain activity in such a way that maintained or even increased performance under conditions of sleep loss was actually observed during more complex tasks when compared with simple ones such as vigilance tasks. It was proposed that this modulated compensatory process is related to increased prefrontal and thalamic activity (19). Importantly, individuals highly differ in compensatory brain activity patterns. The results of the present study suggest that chronotypes differ in cerebral patterns to cope with the passage of time and accumulation of sleep pressure in a regular waking day. Activity was higher in ESs in evening types in a thalamic region, whereas activity was higher in the middle frontal gyrus in morning types during MSs. It is worth noticing that this interaction effect was only found at the most complex task condition, reflecting the highest working memory load in our protocol. Three defining variables were proposed to support activity in the context of total sleep deprivation (23): the state (here, MS vs. ES), the trait (here, morning vs. evening type), and the memory load (N0-, N2-, or N3-back condition in increasing complexity). In the context of this hypothesis (19), we may interpret our data in the perspective that higher task complexity leads to a temporary increase in thalamic-related arousal levels in evening types, which might favor optimal performance in this task condition. Concomitantly, performance in the morning hours in morning types may be supported by increased strategic or attentional recruitment of prefrontal areas.

### Limitations of the study

In this study, we used the most usual N0-, N2-, and N3-back conditions. To include a N1-back condition may have resulted in a more graded design that would have permitted to investigate more precisely the impact of chronotype and time of day on capacity constraints hallmarking working memory at the cerebral level. However, it must be reminded that no interaction effects were found in the N2- vs. N0-back comparison, making it unlikely that supplementary effects would be found in comparison with a N1-back condition. Participants were not systematically trained to the N-back task before performing in the fMRI environment. As a result, two participants had to be excluded in each group because their performance level was far below average. Finally, a significant training effect was observed on performance between the first and the second session. However, half of the participants started with the ES, while the other half started with the MS, to control for order effects and at least partially level out their impact on our results. Retrospectively, we believe that a systematic training to the task would have been appropriate. Finally, as more errors were made in the 3-back compared with the 2-back condition, BOLD variance might be increased in the former. In the same vein, due to the block design, the analysis was not exclusively restricted to correct trials. The application of an event-related design would give interesting supplemental information within this perspective.

## Conclusion

Our results indicate that interindividual differences in sleep–wake regulation should be carefully considered when studying the effect of vigilance states on task-related brain activity. We show here that chronotype-dependent time-of-day fluctuations modulate task complexity-related cerebral activity. Beside, our study highlights the validity of using graded, parametric designs in the investigation of sleep–wake state-induced interindividual differences in the cerebral correlates of cognitive processes.

## Conflict of Interest Statement

The authors declare that the research was conducted in the absence of any commercial or financial relationships that could be construed as a potential conflict of interest.
